# The impact of frailty Screening of Older adults with muLtidisciplinary assessment of those At Risk during emergency hospital attendance on the quality, safety and cost-effectiveness of care (SOLAR): a randomised controlled trial.

**DOI:** 10.1186/s13063-021-05525-w

**Published:** 2021-08-31

**Authors:** Aoife Leahy, Rachel McNamara, Catriona Reddin, Gillian Corey, Ida Carroll, The SOLAR team, Aoife O’Neill, Darragh Flannery, Collette Devlin, Louise Barry, Brian MacCarthy, Niamh Cummins, Elaine Shanahan, Denys Shchetkovsky, Damien Ryan, Margaret O’Connor, Rose Galvin

**Affiliations:** 1 School of Allied Health, Faculty of Education and Health Sciences, Ageing Research Centre, Health Research Institute, University of Limerick, Limerick, Ireland; 2 Department of Ageing and Therapeutics, University Hospital Limerick, Limerick, Ireland; 3 Department of Economics, Kemmy Business School, University of Limerick, Limerick, Ireland; 4 School of Nursing and Midwifery, Faculty of Education and Health Sciences, University of Limerick, Limerick, Ireland; 5 Department of Emergency Medicine, University Hospital Limerick, Limerick, Ireland

**Keywords:** Older People, ISAR, Frailty, Comprehensive geriatric assessment, Emergency department

## Abstract

**Background:**

Older people account for 25% of all Emergency Department (ED) admissions. This is expected to rise with an ageing demographic. Older people often present to the ED with complex medical needs in the setting of multiple comorbidities. Comprehensive Geriatric Assessment (CGA) has been shown to improve outcomes in an inpatient setting but clear evidence of benefit in the ED setting has not been established. It is not feasible to offer this resource-intensive assessment to all older adults in a timely fashion. Screening tools for frailty have been used to identify those at most risk for adverse outcomes following ED visit. The overall aim of this study is to examine the impact of CGA on the quality, safety and cost-effectiveness of care in an undifferentiated population of frail older people with medical complaints who present to the ED and Acute Medical Assessment Unit.

**Methods:**

This will be a parallel 1:1 allocation randomised control trial. All patients who are ≥ 75 years will be screened for frailty using the Identification of Seniors At Risk (ISAR) tool. Those with a score of ≥ 2 on the ISAR will be randomised. The treatment arm will undergo geriatric medicine team-led CGA in the ED or Acute Medical Assessment Unit whereas the non-treatment arm will undergo usual patient care. A dedicated multidisciplinary team of a specialist geriatric medicine doctor, senior physiotherapist, specialist nurse, pharmacist, senior occupational therapist and senior medical social worker will carry out the assessment, as well as interventions that arise from that assessment. Primary outcomes will be the length of stay in the ED or Acute Medical Assessment Unit. Secondary outcomes will include ED re-attendance, re-hospitalisation, functional decline, quality of life and mortality at 30 days and 180 days. These will be determined by telephone consultation and electronic records by a research nurse blinded to group allocation.

**Ethics and dissemination:**

Ethical approval was obtained from the Health Service Executive (HSE) Mid-Western Regional Hospital Research Ethics Committee (088/2020). Our lay dissemination strategy will be developed in collaboration with our Patient and Public Involvement stakeholder panel of older people at the Ageing Research Centre and we will present our findings in peer-reviewed journals and national and international conferences.

**Trial registration:**

ClinicalTrials.gov NCT04629690. Registered on November 16, 2020

## Administrative information

The order of the items has been modified to group similar items (see http://www.equator-network.org/reporting-guidelines/spirit-2013-statement-defining-standard-protocol-items-for-clinical-trials/).

**Table:** 

Title {1}	The impact of frailty Screening of Older adults with muLtidisciplinary assessment of those At Risk during emergency hospital attendance on the quality, safety and cost-effectiveness of care (SOLAR): a randomised controlled trial.
Trial registration {2a and 2b}.	Clinical Trials.gov NCT04629690
Protocol version {3}	07/04/21 Protocol Version 1
Funding {4}	HRB Investigator Led Project Award 2017
Author details {5a}	^1^School of Allied Health, Faculty of Education and Health Sciences, Ageing Research Centre, Health Research Institute, University of Limerick. ^2^Department of Ageing and Therapeutics, University Hospital Limerick ^3^School of Nursing and Midwifery, Faculty of Education and Health Sciences, University of Limerick ^4^Department of Emergency Medicine, University Hospital Limerick ^5^ Department of Economics, Kemmy Business School, University of Limerick
Name and contact information for the trial sponsor {5b}	University of Limerick Castletroy Co. Limerick
Role of sponsor {5c}	The study sponsor played no role in the design, conduct or reporting of the study.

## Introduction

### Background and rationale {6a}

Ireland like many other countries has seen a steady increase in life expectancy and has an ageing population. By 2031, it is forecasted that there will be more than one million older adults in Ireland, representing an increase from 13% (2015) to 20% of our overall population [1]. According to the United Nations, the population over age 65 in Europe and North America is set to increase by 48% between 2019 and 2050 [2]. International evidence demonstrates that older adults account for up to 25% of all Emergency Department (ED) attendances and experience high rates of adverse outcomes following emergency care such as unscheduled ED return, unplanned hospital admission and functional decline [3, 4]. There is a paucity of research conducted to explore how comprehensive geriatric assessment (CGA) implemented in the ED may improve outcomes among those who present to the hospital for emergency care.

CGA is acknowledged as the gold standard of care for the frail older person [5, 6]. Most studies that examine CGA agree that it consists of a medical, social, functional and psychological review and these constructs determine the overall health of an individual. A comprehensive multifaceted approach, thereby, grants the opportunity to create a synergistic management plan for patients with targeted interventions across these broad domains. A Cochrane review [6] has shown that the use of CGA confers quantifiable and reproducible benefits to the health and wellbeing of older people. The value of CGA in various settings and implementation stages has been established. CGA has been shown to potentially reduce mortality and increase home discharge when performed in dedicated geriatric wards as well as when delivered by mobile geriatric units [6]. It has also been investigated as a treatment model in the surgical inpatient unit setting for elective surgical oncology patients and for those recovering from hip fractures [7]. CGA has been shown to have variable outcome benefits in primary and long-term care settings [8]. A systematic review of five randomised controlled trials by Conroy and colleagues (2011) reported that there was no clear evidence of benefit for CGA interventions among older adults in the ED [9]. Mortality [RR 0.92 (95% CI 0.55–1.52)], readmissions [RR 0.95 (95% CI 0.83–1.08)], subsequent institutionalisation, functional ability, quality-of-life or cognition were not statistically significant in the intervention group. The studies primarily investigated single interventions which were heterogenous in nature [9].

The overall aim of this study is to examine the impact of multicomponent CGA on the quality, safety and cost-effectiveness of care in an undifferentiated population of frail older people with medical complaints who present to the ED and Acute Medical Assessment Unit.

### Objectives {7}

1. To implement CGA for frail older adults aged 75 years and over, which will be provided by the SOLAR team including a senior geriatric specialist registrar, senior physiotherapist, senior occupational therapist, senior medical social worker and senior pharmacist in the Emergency Department and Acute Medical Assessment Unit (AMAU) in a University teaching hospital.
2. To examine if early assessment and intervention by the SOLAR team improves the quality, safety and cost-effectiveness of care among frail older adults who present to the ED and AMAU.
3. To conduct a process evaluation of the CGA intervention through focus group interviews with the SOLAR team, representatives from the wider ED and AMAU staff along with patient representatives regarding the feasibility, acceptability, and delivery of the intervention.

### Trial design {8}

This study will be a parallel group randomised control trial with a 1:1 allocation ratio. In order to ensure standardised conduct and reporting, the Consolidated Standards of Reporting Trials (CONSORT) guidelines will be used [10].

## Methods: Participants, interventions and outcomes

### Study setting {9}

The study will take place in the ED and AMAU of a University teaching hospital in the West of Ireland. It serves urban and rural areas of Limerick, Clare and North Tipperary. This is a Model 4 university teaching hospital which caters for the general medical, surgical, and emergency treatment of patients with a catchment area of 400,000 people. Model 4 hospitals have a 24/7 ED which function 365 days a year and are tertiary referral centres for the region.

### Eligibility criteria {10}

The inclusion criteria for participants are adults aged ≥75 years with undifferentiated medical complaints. They must be medically stable as deemed by the treating physician. They must have a score of ≥ 2 on the Identification of Seniors at Risk (ISAR) screening tool and present with a medical complaint [11]. Patients will be screened if they present acutely with Manchester Triage Category 2-5 [12]. Patients with the following presenting complaints will be considered for inclusion: limb problems, falls, unwell adult, back pain, urinary problems, chest pain, shortness of breath, abdominal pain and headache. Patients will be screened for inclusion and recruited between 8 am and 4 pm Monday to Thursday. This is based on the availability of the research nurse. Patients outside of these hours will not be recruited. Exclusion criteria include those under the age of 75 years; a score of less than 2 on the ISAR; acute myocardial infarction or stroke; non-medical problems e.g. surgical or psychiatric issues; medically unstable patients; if neither the patient nor carer can communicate in English sufficiently to complete consent or baseline assessment; and confirmed COVID 19 diagnosis or those with symptoms highly suggestive of COVID 19.

### Who will take informed consent? {26a}

Potential participants will initially be informed of the study by the triage nurse or the treating physician. If participants indicate a willingness to hear more about the study, the dedicated research nurse on the project will be informed. The research nurse will subsequently meet with the potential participant, describe the study in detail and provide potential participants with an information leaflet. Participants will be given time to consider the study and to ask questions. Written informed consent will be obtained by the research nurse if/when the participant indicates their willingness to formally participate in the study. Participants will have the duration of their ED or AMAU stay to consider participation in the study. Participants can withdraw at any stage without any negative implications for their treatment.

It is assumed that adults have the capacity to consent or refuse participation unless it is established by the research nurse that they lack capacity. The research nurse will discuss the study with the patient. Where a patient is able to comprehend, retain and explain back what the study entails to the research nurse, believes the information provided and can weigh up the information to make an informed decision, then capacity will be assumed. Where a patient does not demonstrate these abilities, they will be deemed not to have the capacity to consent to research participation and a proxy consent will be obtained from the next of kin with assent from the patient. The next of kin will be allowed time to consider the study and ask questions on behalf of the patient. This consent may be obtained over the phone due to the current hospital visiting restrictions relating to COVID and there will be documentation of whom the consent was obtained from. Should participants choose to drop out of the study, they will undergo routine clinical practice in the ED or AMAU. As this study is a once-off intervention, should participants drop out after the intervention is completed, they will not receive telephone interviews from the research team.

### Additional consent provisions for collection and use of participant data and biological specimens {26b}

There will be no biological specimens collected for the study.

## Interventions

### Explanation for the choice of comparators {6b}

The comparison group will receive usual care as would be current practice in the ED or AMAU. Currently, there is no dedicated team to perform CGA in the ED and AMAU at UHL with ad hoc allied health assessment available only at the discretion of the referring ED doctor or medical team. This process of usual care will be continued during the study and will be documented.

### Intervention description {11a}

The intervention will comprise initially of a detailed interdisciplinary assessment and intervention by one or more members of the dedicated SOLAR team. The SOLAR team (consisting of a geriatric specialist registrar, specialist geriatric nurse, senior pharmacist, senior physiotherapist, senior occupational therapist, and senior medical social worker) in both ED and AMAU will assess all participants in the intervention group and perform CGA. Potential participants will be approached regarding trial recruitment post ED triage thereby ensuring rapid assessment shortly after hospital arrival by the teams in both AMAU and in ED.

This CGA will include but not be limited to a medical assessment, medication review, nursing assessment, falls assessment, assessment of mobility and stairs, transfers, personal care, activities of daily living (ADLs), social supports, and baseline cognition (Table 1). Members of the SOLAR team will be guided by their clinical expertise and codes of professional practice. Similarly, interventions prescribed by the SOLAR team will be based on subjective and objective assessment of patients and will include medication alterations, lifestyle advice, mobility aids, exercise programmes, ADL equipment, and onward community referral as appropriate. All assessments and interventions will be included in the medical chart of individual participants.

**Table 1:** Components of Comprehensive Geriatric Assessment (Biological, Psychological, Social, Environment and Function)

Biological	Problem list including co-morbid conditions and disease severity Medication review Frailty syndromes (falls, bone health, incontinence) Nutritional assessment
Psychological	Cognition/delirium assessment Mood and anxiety Fears
Social	Basic activities of daily living Gait and balance Activity/exercise status Instrumental activities of daily living
Environment	Home comfort, facilities and safety Use or potential use of tele-health technology, etc. Transport facilities Accessibility to local resources
Functional	Informal support from family or friends Formal community support Eligibility for being offered care resources Social network such as visitors or daytime activities

The control group will have usual care where they undergo assessment by the ED or AMAU physicians with subsequent medical referral if requiring admission.

### Criteria for discontinuing or modifying allocated interventions {11b}

CGA is a dynamic process and may change depending on the patient's presentation. Participation in the study is voluntary. Participants will be free to withdraw consent and leave the study at any time without giving a reason and without affecting their care. Should a patient withdraw consent to participate in the study, we will seek clarification on whether withdrawal is from a particular element of the study, for example, participation in the CGA, questionnaire completion or access to healthcare records. Previously collected anonymised data will still be used in the analyses. In cases where participants do not respond to follow-up assessments, outcome data that do not involve participant contact (e.g. data from hospital database) will continue to be collected in these cases.

No formal interim analyses are planned for the primary or secondary outcomes. A single final analysis is planned after the study is closed to recruitment and follow-up and when the full database has been screened, cleaned and prepared for analysis.

### Strategies to improve adherence to interventions {11c}

A number of strategies have been developed to support adherence to the intervention. A detailed protocol has been created and shared with the SOLAR team. Daily briefings/team meetings are planned to troubleshoot queries or concerns by the team. Training on the procedures around participant consent has been conducted. A comprehensive and detailed delegation log has been developed.

### Relevant concomitant care permitted or prohibited during the trial {11d}

Participants will be under the medical care of their treating physician for the duration of their ED or AMAU stay. All relevant consultant stakeholders will continue to provide clinical governance for the patient as per standard clinical practice. Participants who are admitted as an inpatient will be transferred to a relevant ward following their ED or AMAU stay where their medical care will be transferred to the usual medical team and allied health team as required. There is no care which will be prohibited as part of the trial.

### Provisions for post-trial care {30}

Participants who are discharged from the ED or AMAU to the community setting or nursing home will be discharged to the care of their General Practitioner (GP) and will obtain follow-up with a geriatric service in their catchment area if required. The GP will be informed of their participation in the study. Participants may also be referred to community nursing, allied health professionals or community care teams. A detailed letter describing the CGA intervention and assessment will be sent to the GP and other relevant healthcare professionals for all intervention group participants discharged directly from the ED or AMAU. All other patients will have the usual hospital discharge letter provided on discharge.

### Outcomes{12}

The primary outcome of the study is patient ED length of stay or Patient Experience Time (PET) time, which is recorded as time of arrival at the ED until discharge from ED to home or admission to a ward. For participants in the AMAU, this is defined as the time from arrival at the hospital to discharge from AMAU or admission to a medical ward.

Secondary outcomes include mortality, ED revisit, unplanned hospital visit(s) or nursing home admission within 30 days and 6 months of initial index visit. Healthcare utilisation will also be captured at 30 days (GP visit, Public Health Nurse visit, Allied Health Services) by telephone contact. Follow-up at 30 days and 6 months will be captured by telephone contact by a research nurse who is blinded to group allocation. Patient reported outcomes at 30 days include a global measure of function (Barthel Index) and quality of life survey (EuroQoL-5D EQ-5D) [13, 14]. In addition, patient satisfaction with their index visit will be explored using a patient satisfaction questionnaire. A detailed process evaluation will be completed using a mixed methods approach. The process evaluation will explore stakeholders’ experience of implementing the intervention as well as examining factors that enabled or hindered the delivery of same.

#### Economic evaluation

To examine the cost-effectiveness of the SOLAR intervention, an economic evaluation including both cost-effectiveness and cost-utility analysis will be completed. The evaluation will be conducted in a manner consistent with the guidelines published by the Health and Information Quality Authority in Ireland [15]. The objectives of the evaluation will be to identify, quantify and compare the costs and outcomes of the SOLAR intervention relative to usual care. The costs will reflect those falling on the health and social care system such as return ED visits, inpatient stays, GP visits, medications prescribed and public health nurse visits. The primary and secondary outcomes outlined in the trial will be utilised with regard to intervention effectiveness. The marginal costs and effects of the programme relative to standard care will be calculated through incremental analysis and used to estimate relevant incremental cost-effectiveness ratios. Furthermore, the cost-utility analysis will use patient responses to the EuroQol EQ-5D measurement tool to estimate effectiveness in terms of Quality Adjusted Life Years (QALYs). Budget-impact analyses for the alternative ED patient care approaches will be undertaken. Uncertainty in the estimates will be examined using multivariate sensitivity analyses while cost-effectiveness acceptability curves will also be presented.

#### Process evaluation

The process evaluation will consist of a validated satisfaction survey conducted with study participants at the 30-day follow up call. To further evaluate the trial, one on one telephone interviews will be conducted with trial participants, the SOLAR Team and members of the wider interdisciplinary team to ensure an in-depth evaluation of the process and impact of the trial within the ED and MAU and among study participants. A process evaluation protocol will be formulated to inform and underpin the methodology. Purposive convenience sampling will be utilised.

### Participant timeline {13}

**Fig. 1:**
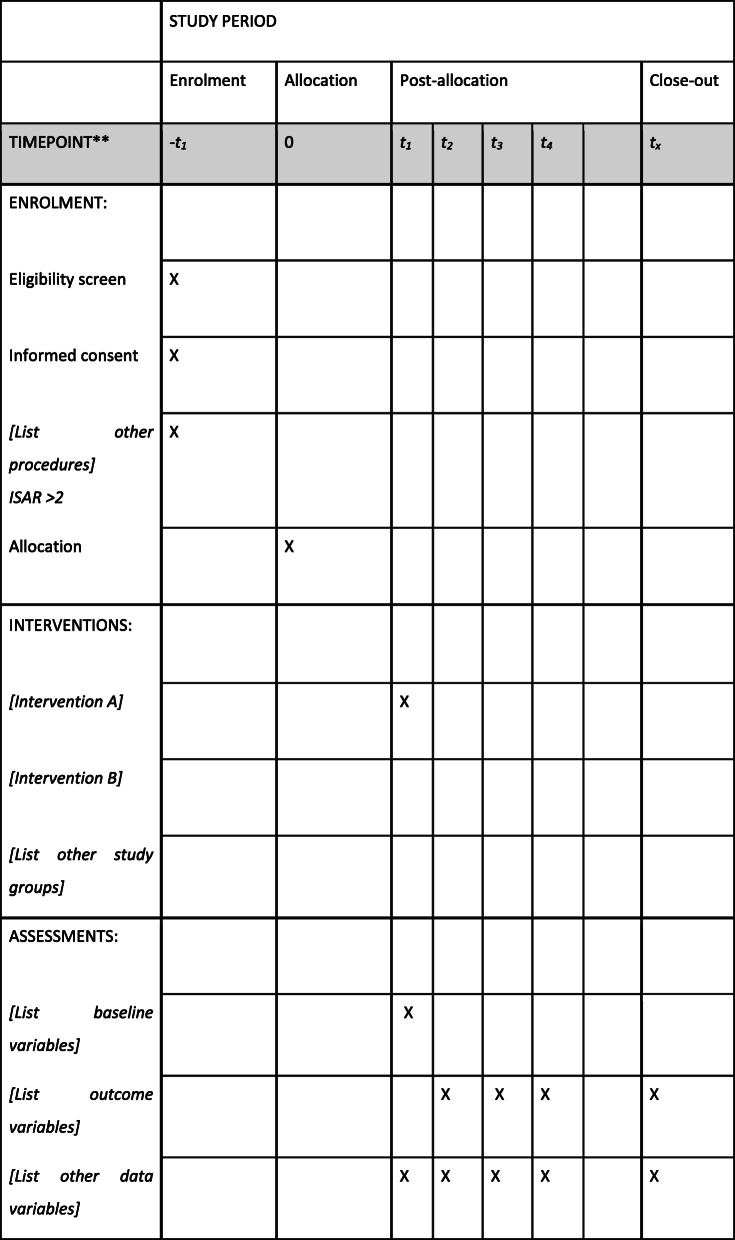
Example template of recommended content for the schedule of enrolment, interventions, and assessments. Recommended content can be displayed using various schematic formats. See SPIRIT 2013 Explanation and Elaboration for examples from protocols. **List specific time points in this row. t1 Assessment. t2 Admission OR discharge. t3 30 day outcomes. t4 180 day outcomes

The participant timeline is shown in Fig. 1

### Sample size {14}

We estimated the sample size based on the primary outcome (ED length of stay) using G*Power version 3.1. Using data from the Patient Experience Time (PET) database employed in the ED at UHL (unpublished data), the average ED length of stay for patients aged 75 and older for the 2019 yearly period was 18.63 h (standard deviation 19.91 h). Estimating a 50% decrease in ED length of stay in the intervention group (mean 9.31 h), and with a 20% attrition rate to follow-up, a sample size of 236 patients (118 in each group) will be required to achieve 90% power with two-tailed tests at an alpha level of 0.05.

### Recruitment {15}

Older adults (≥ 75 years) who screen positive for frailty with ISAR ≥ 2 will be asked if they would like to participate in the study by a dedicated research nurse during their ED or AMAU visit. They will be given an opportunity to read the information leaflet and ask questions before giving informed consent if agreeable. Participation is entirely voluntary.

## Assignment of interventions: allocation

### Sequence generation {16a}

Eligible participants will be block randomised in groups of 50 participants using a computer-generated 1:1 allocation through a Sealed Envelope website (www.sealedenvelope.com).

### Concealment mechanism {16b}

Allocation will be concealed from participants and researchers until both consent has been obtained and baseline evaluations has been performed. After allocation is revealed, the intervention will be organised by the team.

### Implementation {16c}

An independent website (sealedenvelope.com) will be used to generate the allocation sequence. The research nurse will enrol participants and assign participants to interventions once allocation has been revealed.

## Assignment of interventions: blinding

### Who will be blinded {17a}

The outcome assessor will be blinded to group allocation. Clinicians and patients will not be blinded to group allocation. The outcome assessor is not a member of the SOLAR clinical team.

### Procedure for unblinding if needed {17b}

There is no plan to unblind the outcome assessor.

## Data collection and management

### Plans for assessment and collection of outcomes {18a}

All participants will undergo a baseline assessment by the research nurse to include a global measure of function (Barthel Index) and quality of life (EuroQoL-5D [EQ-5D]).

Participants who are allocated to the SOLAR team CGA assessment and intervention will additionally be assessed by one or more members of the interdisciplinary SOLAR team who will complete a detailed clinical assessment including but not limited to managing medical issues and medication review, assessment of mobility and stairs, transfer, personal care, activities of daily living (ADLs), social supports, and cognition. Clinicians will be guided by their clinical expertise and codes of professional practice when completing the assessment.

Similarly, interventions prescribed by the SOLAR team will be based on subjective and objective assessment of patients and will include onward referral as appropriate. All assessments and interventions by the SOLAR team will be included in the medical chart, as is routine clinical practice to inform their medical care. Details of SOLAR assessment and intervention will be collected for the purposes of this study.

Data collection forms will be stored in a data repository and are provided as an appendix to this protocol. Hard copies of the consent form and completed questionnaires will be securely stored in a locked cabinet in a building requiring swipe card access.

### Plans to promote participant retention and complete follow-up {18b}

We will obtain multiple phone numbers for each participant to ensure complete follow-up. If participants are unable to answer, we will ring at a different time of the day and contact all available numbers. We will ask participants if they would like their next of kin to help with follow up phone calls and schedule phone interviews at their convenience. We will also reference hospital information systems during the interview so as to remind patients if they have had a hospital attendance. We will reschedule phone calls if the participant requests this.

### Data management {19}

#### Data entry

All relevant data entered by a research nurse will be stored on Excel and pseudo-anonymised. The key to this anonymisation will be kept by the Primary Investigator (PI). A quality check of 20% of data will be completed by an independent researcher. If there is more than 5% of errors identified across the data entry, all data will be independently checked by the second independent researcher.

#### Data storage

For the life of the study, the pseudo-anonymised data will be stored on an encrypted and password protected electronic data capture system (CASTOR). Each member of the research team designated the task of entering data will have their own unique login and password for this system. This data will be stored on an encrypted computer in a locked office in a building with restricted access.

Hard copies of the study materials will be stored in a locked cabinet in an office with restricted access for 7 years. Anonymised data will be made available on an open access platform (Open Science Framework) after study completion.

### Confidentiality {27}

The confidentiality of the data will be always ensured by the PI and all members of the research team. Identifiable data will not be disclosed to third parties, and no participant’s name will appear in any of the results, as indicated in the participant’s information leaflet. All researchers have completed Good Clinical Practice training.

Each participant in the study will be assigned a numerical code in order to link data collected at baseline to the data collected at follow-up at 30 days and 6 months. Aggregate data will be anonymised.

The research team will ensure anti-virus software is installed and up to date on all devices involved in data entry. All information trafficked from the clinical site to the research database will be pseudo-anonymised with unique study specific subject numbers. Access to the research database is managed by the principal investigator. No personal details or identifying data will be transferred from the hospital site to external sites, where the data will be analysed. Only coded/pseudonymised data will be transferred to external sites.

### Plans for collection, laboratory evaluation and storage of biological specimens for genetic or molecular analysis in this trial/future use {33}

There will be no biological specimens collected in this study.

## Statistical methods

### Statistical methods for primary and secondary outcomes {20a}

Appropriate descriptive statistics will be used to describe the baseline characteristics of study participants. These will include proportions, percentages, ranges, means and standard deviations and medians and interquartile ranges (where data are not normally distributed). We will analyse differences across the two groups for all continuous outcomes (ED length of stay, etc.) by using independent samples *t* tests with 95% confidence intervals. The non-parametric equivalent, the Mann-Whitney test, will be used for skewed data.

Incidence of ED return, unplanned hospital admission, nursing home admission and healthcare utilisation will be explored using crude and adjusted risk ratios with associated 95% confidence intervals using logistic regression. Unit costs will also be applied to resource use data. The total costs of health and social care input will be compared between groups with complete resource utilisation data, using non-parametric bootstrapping (it is anticipated that the data will be highly skewed in terms of distribution). A 5% level of statistical significance will be used throughout the analyses.

### Interim analyses {21b}

There is no plan for an interim analysis.

### Methods for additional analyses (e.g. subgroup analyses) {20b}

There will a subgroup analysis based on discharge location and Clinical Frailty Score [16].

Subgroup analysis exploring the subgroup of control participants who were seen by the OPTIMEND team will be carried out. OPTIMEND is a health and social care professional team who perform certain components of CGA but without specialist geriatric medicine input.

### Methods in analysis to handle protocol non-adherence and any statistical methods to handle missing data {20c}

An intention to treat analysis using linear mixed models will be used to compare outcomes between the intervention and control arm and account for the correlation within-subject over time, while adjusting for differences in participant characteristics at baseline where appropriate. A sensitivity analysis, via multiple imputation, will be used to explore whether adherence to the intervention influences the effect of the intervention on the primary outcome.

### Plans to give access to the full protocol, participant level-data and statistical code {31c}

The anonymised dataset will be stored in a public data repository and supplementary data will be available on request.

## Oversight and monitoring

### Composition of the coordinating centre and trial steering committee {5d}

Given the nature of this trial, The Trial Steering Committee (TSC) will comprise of the PI, key co-applicants, SOLAR team and other key external members of staff involved in the study. Specifically, the TSC will be responsible for: protocol development; obtaining ethical approval; clinical set-up; on-going management; promotion of the study; interpretation and publishing of the results. The TSC will provide overall supervision of the study; in particular monitor study progress, and provide public, clinical, and professional advice, with pre-agreed terms of reference.

### Composition of the data monitoring committee, its role and reporting structure {21a}

For a study of this nature, a separate data monitoring committee is not required. The TSC will serve this function.

### Adverse event reporting and harms {22}

No physical, psychosocial, or financial harm is expected to result from taking part in the study. Participants may decide to withdraw from the study at any time, in which case they have the right to do so without any adverse impact on their current or future care.

Any unexpected adverse events will be documented by the research nurse at 30 days and 6 months and the GP will be notified. Participants will be advised to go to the ED if an adverse event requires urgent assessment.

### Frequency and plans for auditing trial conduct {23}

Trial conduct will be monitored each week by the PI and SOLAR team. A formal meeting will take place in which the team (PI and lead investigator) will discuss the intervention and ensure that there is consistency in the conduct of the intervention. Any deviations from the protocol will be discussed and feedback given to the wider SOLAR team. An Independent research nurse will oversee the data management procedures. The SOLAR trial is also subject to the standard operating procedures of our local clinical research unit (Health Research Institute).

### Plans for communicating important protocol amendments to relevant parties (e.g. trial participants, ethical committees) {25}

All protocol amendments will be notified to the Research Ethics Committee. Any deviations from the published protocol will be documented on the trial registration open platform and in the final manuscript detailing the trial outcome.

### Dissemination plans {31a}

The study has been registered with an authorised registry (ClinicalTrials.gov), according to International Committee of Medical Journal Editors (ICMJE) guidelines, prior to the start of recruitment. Uniform requirements for authorship for manuscripts submitted to medical journals will guide authorship decisions.

The TSC will agree on a publication plan and must be consulted prior to release or publication of any study data. Individual collaborators must not publish data concerning their participants which is directly relevant to the questions posed in the study until the main results of the study have been published. Local collaborators may not have access to study data until after publication of the main study results. A lay summary of the findings will be presented in collaboration with the Patient and Public Involvement stakeholder group of older people at the Ageing Research Centre, University of Limerick.

## Discussion

There have been many interventions introduced in the ED and AMAU which aim to improve outcomes for older frail adults. The interventions and the outcomes assessed have been heterogenous. Preston et al. have described four clusters of interventions which have been highlighted as potentially effective: discharge intervention, staff focused, population focused and intervention component [17]. Overall, none of these systematic reviews have shown a clear benefit of CGA in the ED. Hughes et al. showed that strategies in the ED with multiple interventions may be more beneficial than single interventions alone, however, these multidomain interventions are more difficult and laborious to perform [18]. The SOLAR intervention will be multidisciplinary and interdisciplinary in nature under the governance of a consultant geriatrician and/or ED consultant.

## Trial status

Recruitment commenced on November 9th, 2020, and is due to finish in June 2021. Follow-up will be completed in December 2021.

## Data Availability

Individual data will be available in a data repository once the study analysis has been completed.

## Abbreviations

ED: Emergency department
CGA: Comprehensive geriatric assessment
ISAR: Identification of Seniors At Risk
AMAU: Acute Medical Assessment Unit
HSE: Health Service Executive
PPI: Patient and Public Involvement
UHL: University Hospital Limerick
GP: General Practitioner
PET: Patient Experience Times
QALYs: Quality Adjusted Life Years
PI: Primary Investigator
TSC: Trial Steering Committee
